# Clavicular Gorham-Stout disease: a rare case report and literature review

**DOI:** 10.3389/fendo.2026.1811180

**Published:** 2026-07-02

**Authors:** Bingyan Mao, Qi Peng, Jicai Li, Zuoquan Qin, Shenke Xie, Zhiwei Qin

**Affiliations:** 1 Department of Orthopaedic Surgery, The People′s Hospital of Shimen County (Shimen Hospital of Changsha Medical University), Changde, Hunan, China; 2 Medical Imaging Center, The People′s Hospital of Shimen County (Shimen Hospital of Changsha Medical University), Changde, Hunan, China; 3 Pathology Department, The People′s Hospital of Shimen County (Shimen Hospital of Changsha Medical University), Changde, Hunan, China; 4 Laboratory Department, The People′s Hospital of Shimen County (Shimen Hospital of Changsha Medical University), Changde, Hunan, China

**Keywords:** bone resorption, case report, clavicle, Gorham-Stout disease, rare bone disease

## Abstract

Gorham-Stout disease (GSD) is a rare idiopathic osteolytic disorder, and its diagnosis depends on a comprehensive evaluation of clinical, imaging, and pathological findings, alongside the systematic exclusion of other diseases. We report a case of GSD characterized by progressive clavicular bone destruction and resorption following low-energy trauma. Monitoring biochemical markers related to bone turnover is essential for early diagnosis and disease assessment, which is critical for controlling disease progression. The early administration of osteoclast activity inhibitors, combined with active vitamin D supplementation, can improve prognosis. Treatment plans should be individualized based on the lesion site, the patient’s age, and specific needs. In clinical practice, the routine internal fixation of fractures without a definitive diagnosis may lead to severe consequences.

## Introduction

Gorham-Stout disease (GSD) is a rare condition characterized by painless, progressive bone resorption. Initially described by Jackson in 1838 (1), it was systematically characterized by Gorham and Stout in 1955, primarily manifesting as non-neoplastic vascular or lymphatic hyperplasia accompanied by progressive bone destruction and resorption (2). GSD predominantly affects adolescents and can involve all skeletal structures, including the maxillofacial bones, thorax, pelvis, spine, and long bones of the extremities. To date, approximately 400 cases have been reported worldwide (3), often beginning as a monoskeletal lesion. A poor prognosis is associated with concurrent pleural effusion, chylothorax, spinal involvement, or invasion of surrounding soft tissues and organs (3–7). The disease is characterized by low incidence, heterogeneous clinical presentations, and an unclear pathogenesis, which pose significant challenges for diagnosis and treatment. Unilateral clavicular involvement is extremely rare (8, 9). This article reports a case of GSD with progressive clavicular bone destruction and resorption following low-energy trauma, and discusses its pathogenesis, diagnostic strategies, and treatment options in light of the existing literature, aiming to provide insights for early clinical management.

## Case report

### Patient information and history

A 15-year-old male patient, measuring 170 cm in height and weighing 58 kg with a BMI of 20.1kg/m²; he is in puberty and exhibits growth and development similar to that of his peers. Due to persistent pain lasting one hour following a minor impact to the right shoulder, the patient sought medical attention at another hospital. An X-ray examination revealed a fracture of the right clavicle, which was treated with manual reduction and fixation using an 8-shaped bandage. Five months later, a follow-up X-ray indicated progressive bone resorption of the right clavicle, leading to the patient’s transfer to the People's Hospital of Shimen County (Changde, China) for further diagnosis and treatment on January 27, 2025. The patient denied any history of local skin damage, infection, or other systemic diseases. This study is a retrospective case analysis, and informed consent was obtained from the patient. The text does not contain any personal identifying information. Data collection, diagnosis, and treatment were conducted in accordance with the principles of the Declaration of Helsinki and were approved by the Medical Ethics Committee of Shimen County People’s Hospital (Approval No.: K2025002).

### Clinical findings

The right clavicular region exhibits a flatter contour compared to the contralateral side, lacking the characteristic ‘S’-shaped appearance (**Figure 1**). The overlying skin is devoid of erythema, swelling, or ulceration, and maintains a normal temperature. Palpation reveals that the mid-clavicular and acromial segments of the right clavicle are poorly defined, although partial bone can be palpated at the sternal end. Skin mobility is adequate, with no signs of adhesions or tenderness. Furthermore, the range of motion of the shoulder joint does not demonstrate any significant differences when compared to the contralateral side.

**Figure 1:**
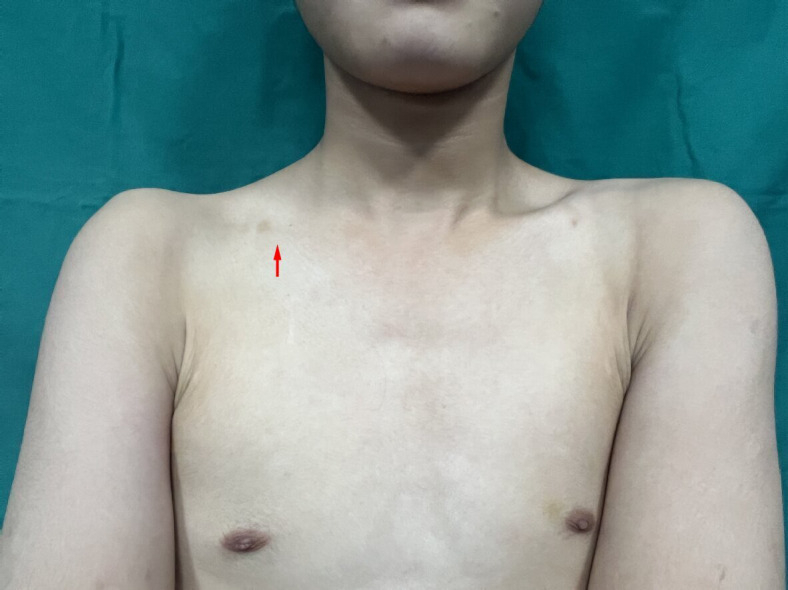
Patient’s chest exterior view. The right clavicular region is flatter than the contralateral side and lacks the typical “S” -shaped appearance(Indicated by the red arrow).

### Diagnostic assessment

Routine blood, urine, and stool tests, along with thyroid and parathyroid function assessments, tumor marker evaluations, and infectious disease screenings, all yielded normal results. However, bone metabolism-related indicators revealed a 25-OH VitD level of 18 ng/mL(reference range: 30–100), the N-Terminal Midfragment of Osteocalcin(N-MID Osteocalcin) measured 23.43 ng/mL(reference range: 4.91–22.31), type I procollagen amino-terminal peptide (PINP) at 339.36 ng/mL(reference range: 15.3–52.7), and β-collagen specific sequence (β-CTx) at 601.92 pg/mL(reference range: <584).

A series of X-ray films revealed progressive osteolysis and resorption of the right clavicle, with the remaining bone gradually thinning into a “spike-like” appearance (**Figure 2**). CT imaging demonstrated osteolysis and disruption of the middle and distal right clavicle, decreased bone density, and preserved adjacent joint spaces, with no pleural effusion or mediastinal abnormalities (**Figure 3**). Furthermore, importing the CT slice DICOM data into Mimics 21.0 software for 3D reconstruction provided a clearer stereoscopic visualization of the morphological characteristics of the right clavicular bone defect (**Figure 4**). MRI indicated thinning and partial loss of the middle and distal right clavicle, with patchy and cystic T2-weighted hypointense signals observed adjacent to the scapula and supraspinatus muscle, accompanied by minimal effusion in the subacromial and coracoid bursae. PET-CT revealed localized bone defects in the middle and distal right clavicle, characterized by thinning and reduced cortical bone mass, decreased density, and soft tissue shadows with mild contrast uptake (SUVmax 2.3) in the defect area. No definitive signs of malignant tumor metabolism were observed systemically.

**Figure 2:**
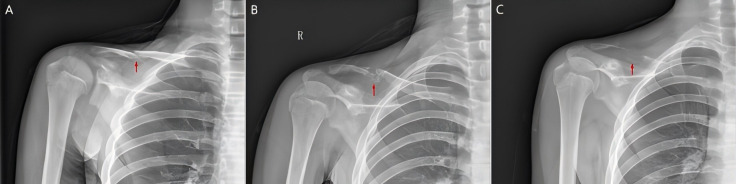
Repeated X-ray examination of the right shoulder. **(A)** shows the image at 1 month post-injury, **(B)** at 2.5 months post-injury, and **(C)** at 5 months post-injury. The red arrow indicates progressive osteolysis and resorption of the right clavicle, with the remaining bone gradually thinning and developing a “spike-like” appearance.

**Figure 3:**
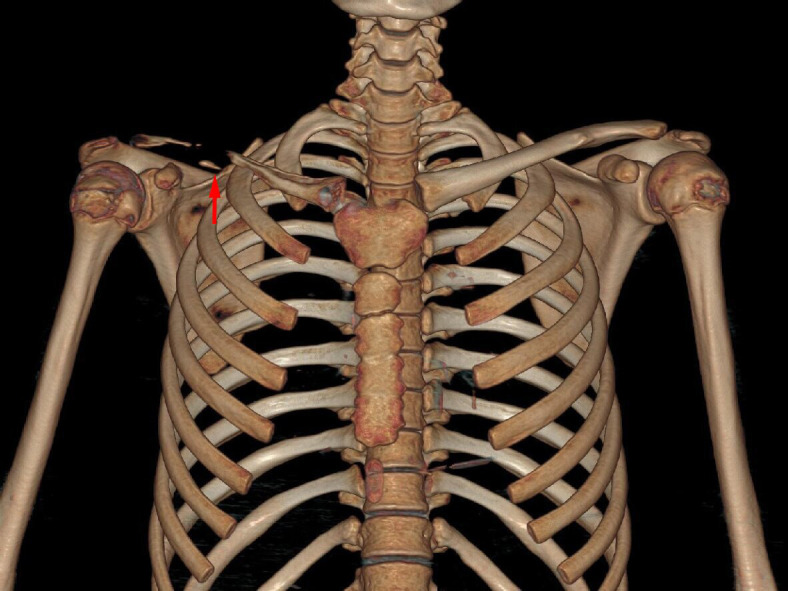
Three-dimensional bone reconstruction images from chest CT. The red arrow indicates the lesion in the right clavicle.

**Figure 4:**
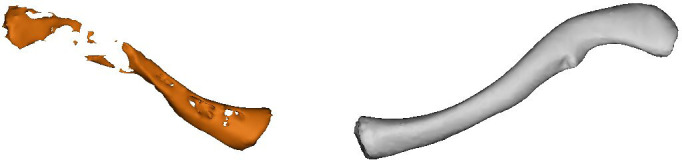
3D reconstruction of the clavicle. Three-dimensional reconstruction provides clearer and more three-dimensional visualization of the morphological features of the clavicular bone defect on the affected side.

Pathological examination of the surgical biopsy revealed evidence of osteonecrosis at the margin of the submitted bone tissue. This finding was accompanied by local vascular dilation between the trabeculae, mild proliferation of fibrous tissue, and infiltration by lymphocytes, plasma cells, and a small number of neutrophils. No tumor cells were observed (**Figure 5**).

**Figure 5:**
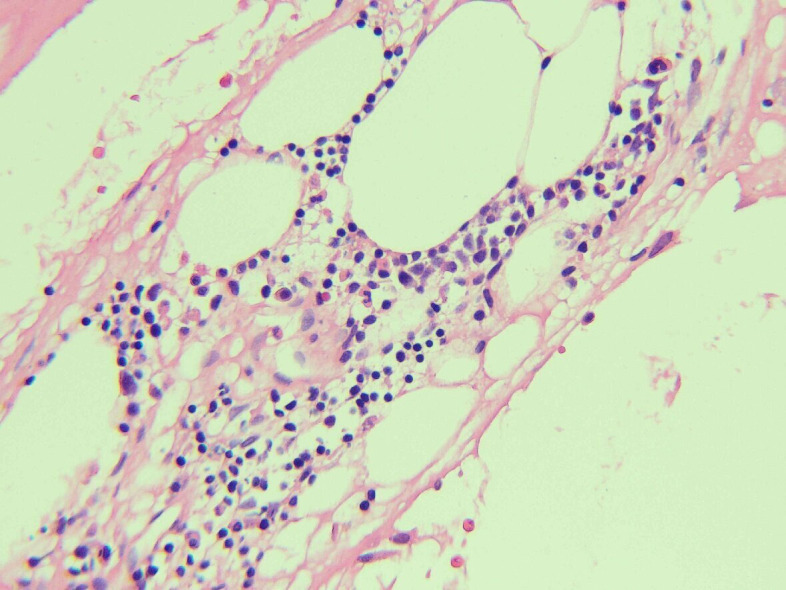
Histopathological analysis (Hematoxylin & Eosin stain 40×10). Local trabecular vasodilation, mild fibrous tissue proliferation, with infiltration of lymphocytes, plasma cells, and a small number of neutrophils; no tumor cells observed.

### Diagnosis and treatment follow-up

The patient was ultimately diagnosed with right-sided clavicular Gorham-Stout disease, accompanied by vitamin D deficiency. Currently, clavicular osteolysis has not significantly affected shoulder joint function, and the epiphysis remains incompletely closed, indicating potential for further growth. Given the CT findings that show no pleural effusion, as well as the MRI and pathology results revealing no significant vascular or lymphatic hyperplasia, the decision was made to temporarily avoid surgery. Instead, an oral treatment regimen was initiated, consisting of Risedronate Sodium Tablets combined with active vitamin D and calcium supplements. After one year of follow-up, the condition remained stable, with no further clavicular dissolution and essentially normal shoulder joint function. Long-term efficacy will require continued monitoring.

## Discussion

Gorham-Stout disease (GSD) is a rare disorder characterized by the abnormal proliferation of benign lymphatic vessels within and around bones, resulting in progressive bone resorption (10–12). A recent systematic review of 206 patients revealed a bimodal age distribution for GSD onset, with approximately 50.7% of cases beginning in childhood (<18 years). Males are slightly more frequently affected than females. Among the patients studied, 77.2% exhibit polyostotic involvement, while monoskeletal lesions predominantly occur in the mandible. The most frequently affected sites overall include the spine, ribs, hip and femur (3). Clinical manifestations are directly related to the affected site, with localized pain being the most common complaint, followed by pathological fractures and localized swelling. When lesions involve the thoracic vertebrae, ribs, or scapula, chylothorax may occur, which serves as a significant prognostic indicator of poor outcomes and markedly increases the risk of mortality (4, 11, 13). Involvement of the skull base can lead to cerebrospinal fluid leakage and recurrent meningitis (14–17), while spinal involvement may result in vertebral fractures and spinal cord compression, potentially leading to paraplegia (18, 19). Additionally, GSD affects not only skeletal lesions but may also involve visceral organs such as the lungs, liver, and spleen, as well as cutaneous soft tissues (3, 20–23).

Rossi M et al. conducted bone biopsies, serological, cytological, and molecular analyses on seven patients with Gorham-Stout disease (GSD) and found significant fibrosis, lymphovascular proliferation, increased osteoclasts, enlarged osteocytic pits in bone tissue, elevated serum levels of ICTP, sclerostin, IL-6, and VEGF-A; enhanced osteoclastic differentiation and bone resorption capacity, impaired osteoblastic mineralization function, and increased RANKL/OPG ratio. Further experiments using patient serum to treat healthy donor cells demonstrated its ability to promote osteoclast generation and bone resorption, inhibit osteoblastic mineralization, and stimulate endothelial cell tubulation. For the first time, a comprehensive analysis was systematically conducted at tissue, cellular, molecular, and humoral levels to elucidate the mechanisms underlying bone loss in GSD, revealing that multiple factors—including enhanced osteoclastic activity, impaired osteoblastic mineralization, and systemic cytokine imbalances (e.g., IL-6, VEGF-A, sclerostin)—collectively drive bone loss, suggesting potential for multi-target therapy (24). Other studies have shown that excessive secretion of osteoclast-promoting cytokines leads to osteoclast hyperactivation and cytokine storms, thereby accelerating bone resorption (13, 14, 25, 26). Recent research has confirmed that abnormal activation of the PI3K/Akt/mTOR pathway is a key driver of endothelial cell proliferation (27). Additionally, KRAS mutations detected in tissues or blood samples of some GSD patients impair the inhibition of downstream proliferative pathways (including the mTOR pathway), further promoting lymphatic or vascular proliferation. These alterations in the local microenvironment, characterized by cytokine secretion and potential direct intercellular communication, directly lead to excessive activation of osteoclasts (28, 29), creating a vicious cycle.

The diagnosis of Gorham-Stout disease (GSD) primarily relies on an exclusionary process. A clinical diagnosis can only be confirmed after thoroughly ruling out infections, malignancies, metabolic bone disorders (such as hyperparathyroidism), and other hereditary osteolytic syndromes (3). Currently, the comprehensive clinical-imaging-pathological criteria proposed by Heffez et al. (30) are widely referenced. The core of these criteria is the confirmation of progressive bone resorption accompanied by lymphangiogenesis or angiogenesis, along with the absence of cellular atypia or other definitive etiologies (10). Zhou Zilong et al. analyzed that among the eight diagnostic criteria for GSD proposed by Heffez et al., the criterion of “absence of visceral involvement” is no longer applicable, while the remaining seven criteria continue to assist in the diagnosis of GSD (3).

Due to the absence of specific biochemical or genetic markers, routine laboratory tests often yield nonspecific results. Most patients exhibit normal levels of serum calcium, phosphorus, and parathyroid hormone; however, during periods of disease activity—particularly when pathological fractures occur—serum alkaline phosphatase (ALP) levels may show mild elevations. More sensitive biochemical markers that reflect bone turnover, such as PINP and β-CTX, can be elevated in some patients with active osteolytic disease, serving as potential tools for early diagnosis, monitoring disease progression, and evaluating treatment responses (31–33). In this case, we incorporated the measurement of biochemical markers of bone turnover into routine examinations. Significant increases in PINP and N-MID osteocalcin indicate extremely high osteoblastic activity; the marked elevation of β-CTX demonstrates active osteoclast activity. This suggests that both bone destruction and repair processes are highly active. In response to extensive bone destruction, the body attempts to initiate repair. Osteoblasts are extensively recruited and activated, striving to synthesize new bone matrix to fill the dissolved bone defects. Additionally, in Gorham-Stout disease, abnormal lymphatic or vascular tissues infiltrating the bone secrete large amounts of cytokines and growth factors, including vascular endothelial growth factor, interleukin-6, and prostaglandin E2 (14). These factors are potent activators of osteoclasts, recruiting and significantly activating them, which leads to a sharp increase in local bone absorption. This disruption severely affects the balance, as abnormal lymphatic or vascular tissues continuously erode. Consequently, the newly formed bone matrix may not have sufficient time to fully mineralize, mature, and consolidate before being destroyed in the subsequent round of erosion. Ultimately, this renders the repair process ineffective and futile.

The primary function of vitamin D is to enhance intestinal calcium absorption, maintain normal serum calcium and phosphorus levels, and provide essential materials for bone mineralization. In this case, the patient’s laboratory test revealed a 25-OH VitD level of 18 ng/ml, indicating vitamin D deficiency. This deficiency leads to inadequate calcium absorption, resulting in decreased serum calcium levels, which in turn stimulates increased secretion of parathyroid hormone (PTH). PTH is a potent factor that activates osteoclasts. Consequently, vitamin D deficiency may exacerbate the already heightened osteoclast activity, further intensifying bone resorption. Additionally, vitamin D deficiency impairs the mineralization process of osteoblasts (34). Active vitamin D can inhibit the production of pro-inflammatory cytokines, regulate T lymphocyte function, and maintain immune tolerance (35–37). As a result, vitamin D deficiency may weaken immune regulatory functions, complicating the body’s ability to suppress localized, abnormal inflammatory responses and vascular or lymphatic hyperplasia, potentially creating an unfavorable environment for disease onset or progression (38). A state of vitamin D deficiency inevitably compromises bone stability and may influence disease progression through immunological pathways. Therefore, in the diagnosis and treatment of Gorham-Stout disease, the detection of biochemical markers of bone turnover and active vitamin D is an essential component of definitive diagnosis and assessment of disease activity.

The selection of treatment for Gorham-Stout disease requires a highly individualized approach, taking into account the patient’s age, the skeletal sites affected, the functional impact of the disease, and any associated complications. Current primary treatment modalities include pharmacological intervention, radiotherapy, and surgical procedures (11). Pharmacotherapy is foundational (39, 40), with bisphosphonates recognized as the first-line treatment due to their capacity to inhibit osteoclast activity, thereby effectively alleviating bone pain and delaying the progression of osteolysis (39). Interferon-α2b has shown stable disease control, particularly in pediatric patients, attributed to its anti-angiogenic properties. Among the most innovative agents is the mTOR inhibitor sirolimus, which targets and inhibits lymphangiogenesis, resulting in substantial clinical and radiological improvements in patients experiencing life-threatening chylothorax or extensive multi-bone involvement (39, 41, 42). Radiotherapy is primarily indicated for locally advanced lesions that are refractory to pharmacotherapy or for patients at high surgical risk. German Cooperative Group on Radiotherapy for Benign Diseases study demonstrated that radiotherapy can effectively halt disease progression in 80% of GS cases, with a recommended total dose of 30–45 Gy administered in fractions of 1.8-2.0 Gy to inhibit abnormal angiogenesis and stabilize bone destruction (43). Surgical intervention is mainly reserved for addressing complications such as internal fixation of pathological fractures, joint replacement (e.g., shoulder or hip), spinal fusion for spinal instability, or repair of skull defects (12, 16, 19). However, the timing of surgery is critical; performing it during active osteolysis may result in implant failure or absorption of bone grafts. All treatments necessitate a careful evaluation of their associated risks and benefits. Bisphosphonates require vigilance due to rare complications such as mandibular necrosis and nephrotoxicity; sirolimus may induce immunosuppression, stomatitis, and metabolic abnormalities; radiotherapy carries long-term risks of secondary malignancies; and surgical interventions are associated with potential infections, hemorrhage, and procedural failure. In this case, the lesion was confined to the clavicle, with minimal impact on shoulder joint function. Given the patient’s young age and potential for growth, MRI revealed localized vascular and lymphatic proliferation, while pathological examination showed no extensive vascular or lymphatic hyperplasia. Consequently, the patient received oral treatment with reserpine combined with active vitamin D and calcium to control disease progression, prevent spread to surrounding bones, and minimize long-term functional impairment. For future clavicular reconstruction, options include 3D-printed clavicular prostheses (44) or free fibular flap transplantation (45, 46). Through a literature review, we have developed a diagnostic and therapeutic strategy for GSD. When symptoms of bone resorption and dissolution occur, GSD should be considered (**Figure 6**).

**Figure 6:**
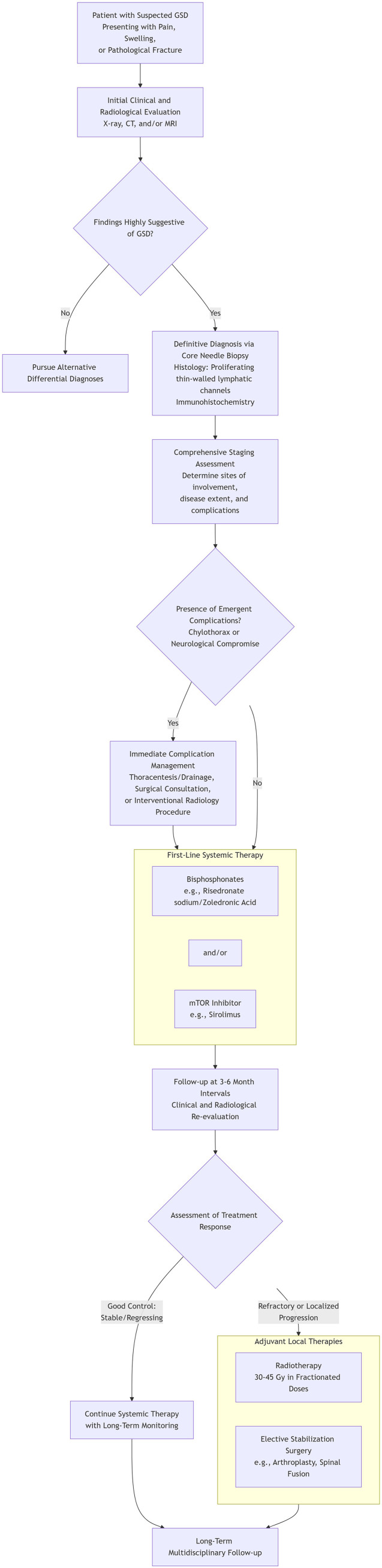
Diagnosis and treatment strategy diagram of Gorham-Stout disease.

## Conclusion

Gorham-Stout disease is a rare osteolytic disorder that requires a comprehensive diagnostic approach, which integrates clinical assessment, imaging studies, and pathological evaluation, while strictly excluding other conditions. Treatment should be individualized, considering the patient’s age, lesion location, and functional mobility. In cases involving osteolytic lesions of unknown etiology, definitive internal fixation surgery should be performed cautiously. If surgery has been conducted, anti-osteolytic or targeted therapy should be initiated as early as possible to improve prognosis. As research into molecular mechanisms advances, more precise therapies are expected to emerge in the future, thereby enhancing patients’ quality of life.

## Data Availability

The raw data supporting the conclusions of this article will be made available by the authors, without undue reservation.
